# Oroxin B alleviates osteoarthritis through anti-inflammation and inhibition of PI3K/AKT/mTOR signaling pathway and enhancement of autophagy

**DOI:** 10.3389/fendo.2022.1060721

**Published:** 2022-12-01

**Authors:** Rui Lu, Zhiyi He, Weikai Zhang, Yingguang Wang, Peng Cheng, Zhengtao Lv, Xuefeng Yuan, Fengjing Guo, Hongbo You, An-min Chen, Weihua Hu

**Affiliations:** ^1^ Department of Orthopedics, Tongji Hospital, Tongji Medical College, Huazhong University of Science and Technology, Wuhan, China; ^2^ Department of Traumatology, Tongji Hospital, Tongji Medical College, Huazhong University of Science and Technology, Wuhan, China

**Keywords:** osteoarthritis, oroxin B, chondrocytes, inflammation, PI3K/AKT/mTOR, autophagy

## Abstract

**Background:**

Osteoarthritis (OA) is a common aging-related degenerative joint disease with chronic inflammation as its possible pathogenesis. Oroxin B (OB), a flavonoid isolated from traditional Chinese herbal medicine, possesses anti-inflammation properties which may be involved in regulating the pathogenesis of OA, but its mechanism has not been elucidated. Our study was the first to explore the potential chondroprotective effect and elucidate the underlying mechanism of OB in OA.

**Methods:**

*In vitro*, primary mice chondrocytes were stimulated with IL-1β along with or without the administration of OB or autophagy inhibitor 3-methyladenine (3-MA). Cell viability assay was measured with a cell counting kit-8 (CCK-8). The phenotypes of anabolic-related (Aggrecan and Collagen II), catabolic-related (MMP3, MMP13, and ADAMTS5), inflammation-related (iNOS, COX-2, TNF-α, IL-6, and IL-1β), and markers of related signaling pathways in chondrocytes with different treatment were detected through western blot, RT-qPCR, and immunofluorescent staining. *In vivo*, the destabilized medial meniscus (DMM) operation was performed to establish the OA mice model. After knee intra-articular injection with OB for 8 weeks, the mice’s knee joints were obtained for subsequent histological staining and analysis.

**Results:**

OB reversed the expression level of anabolic-related proteins (Aggrecan and Collagen II) and catabolic-related (MMP3, MMP13, and ADAMTS5) in IL-1β-induced chondrocytes. Mechanistically, OB suppressed the inflammatory response stimulated by IL-1β, as the inflammation-related (iNOS, COX-2, TNF-α, IL-6, and IL-1β) markers were downregulated after the administration of OB in IL-1β-induced chondrocytes. Besides, the activation of PI3K/AKT/mTOR signaling pathway induced by IL-1β could be inhibited by OB. Additionally, the autophagy process impaired by IL-1β could be rescued by OB. What’s more, the introduction of 3-MA to specifically inhibit the autophagic process impairs the protective effect of OB on cartilage. *In vivo*, histological staining revealed that intra-articular injection of OB attenuated the cartilage degradation, as well as reversed the expression level of anabolic and catabolic-related proteins such as Aggrecan, Collagen II, and MMP13 induced in DMM-induced OA models.

**Conclusions:**

The study verified that OB exhibited the chondroprotective effect by anti-inflammatory, inhibiting the PI3K/AKT/mTOR signaling pathway, and enhancing the autophagy process, indicating that OB might be a promising agent for the treatment of OA.

## Introduction

Osteoarthritis (OA) is a common degenerative joint disease with pain and dysfunction as the main symptoms and cartilage destruction as the basic pathological feature, and occurs mainly in the elderly (1). Risk factors for OA include age, trauma, joint infection, and obesity, but the exact pathogenic mechanism remains unknown (2). In recent years, chronic inflammatory response is considered to be the main pathogenic mechanism of OA (3). Interleukin-1β (IL-1β), which induces inflammatory response and is closely related to the occurrence and progression of OA, is a commonly used cytokine for establishing OA cell models *in vitro* experiment (4). Studies illustrated that IL-1β can induce the decreased expression of anabolic-related proteins, such as Aggrecan and type II collagen (Collagen II), also trigger the overexpression of catabolic-related proteins, such as matrix metallopeptidase 3 (MMP3), matrix metallopeptidase 13 (MMP13), and a disintegrin and metalloproteinase with thrombospondin motifs 5 (ADAMTS5). Then an imbalance of cellular anabolism and catabolism will arise, triggering the degradation of the extracellular matrix (ECM) of the cartilage and the occurrence of OA (5). Besides, IL-1β can promote the release of a large number of pro-inflammatory cytokines, which also contribute to the degradation of cartilage. Therefore, basic research and clinical practice have proved that anti-inflammatory treatment is considered an effective way to deal with OA (6, 7).

Oroxin B (OB), a flavonoid isolated from traditional Chinese herbal medicine *Oroxylum indicum (L.) Vent (* 8). Previous studies have revealed that OB possesses anti-inflammation and anti-tumor properties. It has been demonstrated that OB could downregulate the protein expression of cyclooxygenase-2 (COX-2); phosphoinositide 3-kinase (PI3K), and phospho-AKT (p-AKT) (9). OB also prevented ovariectomy (OVX)-induced bone loss by suppressing osteoclast formation and activity through abrogating the increased phosphorylation level of the mitogen-activated protein kinase (MAPK) and nuclear factor-kappa B (NF-κB) signal pathways (10). The activation of the MAPK and NF-κB signaling pathways which closely related to OA causes the release of inflammatory factors and leads to a reduction in cartilage anabolism and an increase in catabolism, which in turn triggers the degradation of the ECM (11). In addition, the PI3K/AKT/mTOR signaling pathway is a signaling pathway closely related to both inflammatory signaling and autophagy process (12). Its activation also leads to the release of inflammatory signals and impairs the autophagy process, all of which aggravate OA progression (13). However, the effect of OB in OA is unknown. Thus, we designed the experiment to explore the protect role of OB in OA and elucidate the potential mechanism.

## Materials and methods

### Reagents

Oroxin B (OB, HY-N1435, purity: 99.71%, CAS number: 114482-86-9), Dimethyl sulfoxide (DMSO, HY-Y0320, purity: ≥99.0%, CAS number: 67-68-5), and autophagy inhibitor 3-methyladenine (3-MA, HY-19312, purity: 99.83%, CAS number: 5142-23-4) were acquired from MedChemExpress (Monmouth Junction, NJ, USA). Recombinant mouse IL-1β was purchased from R&D systems (Minneapolis, USA). Boster (Wuhan, Hubei, China) provided the RIPA lysis buffer, protease inhibitors, phosphatase inhibitors, BCA protein assay kit, protein loading buffer, rabbit/mouse secondary antibodies, and DAPI reagent. The ECL chemiluminescent substrate was obtained from Bio-Rad (Hercules, CA, USA). The biotechnology of Biosharp Life Sciences (Hefei, Anhui, China) provided the Trypsin (BL527A) and collagenase II (BS164). DMEM/F12 medium culture was got from Hyclone (Logan, UT, USA). Foetal bovine serum (FBS) was purchased from BioInd (Biological Industries, Israel). Omega Bio-tek (USA) provided the RNA extraction kit (R6834-01). Yeasen (Shanghai, China) provided the kits for complementary DNA (cDNA) synthesis (11141ES60) and quantitative real-time polymerase chain reaction (RT-qPCR) (11201es08).

### Harvest and culture of chondrocytes

The primary chondrocytes were harvested from the knee joints of five days old C57BL/6 male mice. After disinfecting, the knee joints were separated, then the transparent knee cartilage particles were collected from the joint capsule. After removing the synovium, fat, tendon and other tissues around the cartilage particles, the cartilage particles are shredded and then digested with 0.25% trypsin for 30 min at 37°C. Then discarded the trypsin, the chondrocyte samples were digested with 0.2% collagenase II for 4-6 h in a hybridization oven at 37°C. Afterwards, chondrocytes were resuspended after removing the collagenase II, and cultured in DMEM/F12 medium supplemented with 10% FBS in an incubator with 5% CO_2_ at 37 °C. Cells in the first and second passages were used for subsequent experiments.

### Chondrocytes viability measurement

The viability of chondrocytes treated with IL-1β or OB or 3-MA were determined by a cell counting kit-8 (CCK-8, Boster, Wuhan, China). Chondrocytes were seed into 96-well plates (5×10^3^ cells per well) for 24 h. Then cells were cultured with different treatments. Afterwards, CCK-8 solution was applied following the manufacturer’s instructions, the cells’ optical density value which could reflect the cell viability was measured at the wavelength of 450 nm with a microplate reader (BioTek, USA).

### Western blot analysis

Chondrocytes with different pretreatment were lysed with RIPA buffer containing 1% phosphatase and protease inhibitors on the ice for 15 min. Then the cells and lysate mixture were further lysed with an ultrasonic disruptor. Centrifuged the mixture samples and collected supernatant as the protein samples. BCA protein assay kit was applied to measure the protein concentration with the help of a microplate reader. Denatured the protein samples which were mixed with protein loading buffer at 100°C for 5 min. Protein samples of 15 µg were separated by SDS-PAGE gels and electrotransfered to PVDF membranes (Millipore, USA). Afterwards, the membranes were blocked with 5% skim milk at room temperature for 1 h, next incubated with corresponding primary antibodies (**Table 1**) at 4°C for more than 14-16 h, then immunoblotted with secondary antibody of corresponding species at room temperature for 1 h. Finally, the membrane bands coated with chemiluminescent substrate were visualized with an exposure system (Bio-Rad, Hercules, CA, USA).

**Table 1:** Primary antibodies for western bolt.

Primary antibody	Catalog number	Dilution ratio	Source
GAPDH	60004-1-Ig	1:50,000	Proteintech Group, Wuhan, Hubei, China
Aggrecan	13880-1-AP	1:1,000	Proteintech Group, Wuhan, Hubei, China
Collagen II	28459-1-AP	1:1,000	Proteintech Group, Wuhan, Hubei, China
MMP3	17873-1-AP	1:500	Proteintech Group, Wuhan, Hubei, China
TNF-α	17590-1-AP	1:500	Proteintech Group, Wuhan, Hubei, China
MMP13	18165-1-AP	1:1,000	Proteintech Group, Wuhan, Hubei, China
TGF-β1	21898-1-AP	1:1,000	Proteintech Group, Wuhan, Hubei, China
p-AKT	66444-1-Ig	1:2,000	Proteintech Group, Wuhan, Hubei, China
AKT	60203-2-Ig	1:5,000	Proteintech Group, Wuhan, Hubei, China
p-mTOR	67778-1-Ig	1:2,000	Proteintech Group, Wuhan, Hubei, China
mTOR	66888-1-Ig	1:5,000	Proteintech Group, Wuhan, Hubei, China
P62 p-PI3K PI3K	18420-1-AP #4228 #4249	1:1,000 1:1,000 1:1,000	Proteintech Group, Wuhan, Hubei, China Cell Signaling Technology, Beverly, MA, USA Cell Signaling Technology, Beverly, MA, USA
Atg3	#3415	1:1,000	Cell Signaling Technology, Beverly, MA, USA
Atg5	#12994	1:1,000	Cell Signaling Technology, Beverly, MA, USA
Atg7	#8558	1:1,000	Cell Signaling Technology, Beverly, MA, USA
Beclin-1	#3495	1:1,000	Cell Signaling Technology, Beverly, MA, USA
LC3 I/II	#12741	1:1,000	Cell Signaling Technology, Beverly, MA, USA
iNOS	#13120	1:1,000	Cell Signaling Technology, Beverly, MA, USA
COX-2	# 12282	1:1,000	Cell Signaling Technology, Beverly, MA, USA
IL-1β	#12242	1:1,000	Cell Signaling Technology, Beverly, MA, USA
IL-6	A14687	1:500	ABclonal, Wuhan, Hubei, China

### RT-qPCR analysis

Total RNA of chondrocytes was extracted using a RNA isolation kit, then the RNA samples were reverse transcribed to cDNA using a cDNA synthesis kit. Afterwards, the cDNA was amplified with a RT-qPCR kit. The primer sequences of the genes were used as follows: *MMP3* (Forward: 5’-ACTCCCTGGGACTCTACCAC-3’; Reverse:5’-GGTACCACGAGGACATCAGG-3’),*MMP13*(Forward:5’-GATGGACCTTCTGGTCTTCT-3’; Reverse: 5’-GCTCATGGGCAGCAACAATA-3’), *ADAMTS5* (Forward:5’-GCAAAGTGGGCTACCTTGTC-3’;Reverse:5’-GTTTCTACAGAGGCACCGTG-3’), *GAPDH* (Forward: 5’-TGTTTCCTCGTCCCGTAGAC-3’; Reverse: 5’-GTTGAGGTCAATGAAGGGGTC-3’). The mRNA expression levels of target genes were normalized to *GAPDH* and calculated with a comparative2 ^−ΔΔCt^ method.

### Immunofluorescence staining

The chondrocytes seeded into 24 well plates (2×10^4^ cells per well). After received different treatment, the cells were fixed with 4% paraformaldehyde (Servicebio, Wuhan, China) for 15min and permeabilized with 0.2% Triton X-100 (BioFroxx, Germany) for 5 min. After that, cells were followed by blocking with 5% bovine serum albumin (BSA, BioFroxx, Germany) at room temperature for 1 h, incubated with primary antibodies (**Table 2**) at 4°C for more than 14-16 h, immunoblotted with fluorescent secondary antibody of corresponding species at room temperature in the dark for 1 h, and stained with DAPI reagent for 10 min. Finally, removed excess dye reagent and washed the cells, the fluorescence pictures were obtained by using a scanning microscope (Evos Fl Auto, Life Technologies, USA).

**Table 2:** Primary antibodies for immunofluorescence cell staining.

Primary antibody	Catalog number	Dilution ratio	Source
Aggrecan	13880-1-AP	1:100	Proteintech Group, Wuhan, Hubei, China
Collagen II	28459-1-AP	1:200	Proteintech Group, Wuhan, Hubei, China
MMP13	18165-1-AP	1:50	Proteintech Group, Wuhan, Hubei, China

### GFP-RFP-LC3 adenovirus transfection

For evaluating the strength of autophagic flux among the groups, mice chondrocytes were transfected with the tandem GFP-RFP-LC3 adenovirus (HanBio Technology, Shanghai, China) under the manufacturer’s guideline. Afterwards, IL-1β and OB were added to the cells of different groups to clarify their impact on autophagic flow. Then a confocal microscope (Nikon America Inc., Melville, NY) was applied to obtain the autophagy flow pictures in which the yellow puncta represented the autophagosomes, while the red puncta reflected the autolysosomes.

### Mice OA model generation and treatment

For *in vivo* experiments, the destabilized medial meniscus (DMM) method was applied in the right knee joints of mice to establish to the OA model. Thirty-two C57BL/6 male mice of 8 weeks old were conducted under specific pathogen-free grade conditions. The *in vivo* experiment was in accordance with the guidelines of the International Guiding Principles for Animal Research and approved by the Ethics Committee on Animal Experimentation of Tongji Hospital, Tongji Medical College, Huazhong University of Science and Technology. The mice were randomly and equally divided into 4 groups, namely the SHAM group (n=8), the SHAM + OB group (n=8), the DMM group (n=8), and the DMM + OB group (n=8). In brief, the SHAM and SHAM + OB groups were underwent the sham surgery (only underwent joint capsulotomy), while the DMM and DMM + OB group mice were underwent the DMM operation. One week after the above operations were done, treatment of intra-articular injections of the knee joint twice a week for 8 weeks was performed. Vehicle (30% PEG300, 5% DMSO, and ddH2O) of 10 μl was injected into the knee joints of mice in the SHAM and DMM groups, while OB (160 μM) of 10 μl was applied in the SHAM + OB group and DMM + OB group.

### Histological staining of mice knee samples

The obtained knee joints were fixed with 4% paraformaldehyde, decalcified, embedded in paraffin, and then cut into 5-μm sections for further experiments. Then knee samples were stained with hematoxylin plus eosin (H&E), safranin O/fast green (SOFG), and immunohistochemistry (IHC). Based on the localization of the protein, such as in the cytoplasm or nucleus, the cells stained brown in the region of the target protein were defined as positive cells, and then positive chondrocytes were calculated using the counting function in ImageJ (Software version 1.53a, National Institutes of Health, USA). The primary antibodies used in IHC staining are listed in **Table 3**.

**Table 3:** Primary antibodies for IHC staining.

Primary antibody	Catalog number	Dilution ratio	Source
Aggrecan	13880-1-AP	1:200	Proteintech Group, Wuhan, Hubei, China
Collagen II	28459-1-AP	1:800	Proteintech Group, Wuhan, Hubei, China
MMP13	18165-1-AP	1:100	Proteintech Group, Wuhan, Hubei, China

### Statistical analysis

The experiment data were repeated at least three times and presented as mean ± standard deviation (SD). One-way analysis of variance (ANOVA) followed by Dunnett’s *post hoc* test was applied to analyze data among multiple comparisons. Besides, Kruskal-Wallis H test was used to analyzed the nonparametric data (OARSI scores). Data analysis was done using GraphPad Prism version 8.4.0 (GraphPad Software, California, USA). Statistical significance was considered as *p < 0.05, **p < 0.01, ***p < 0.001.

## Results

### Viability of chondrocytes were not affected by IL-1β/OB/3-MA

**Figure 1:**
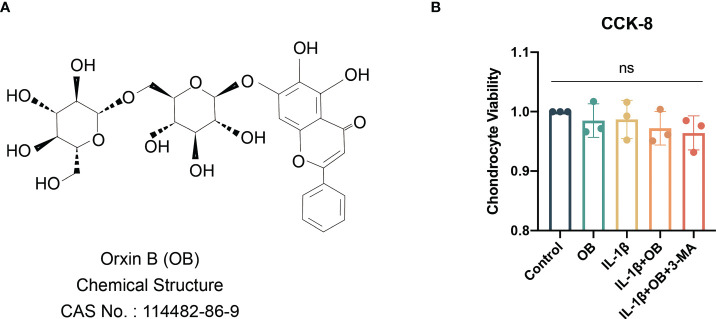
Viability of chondrocytes were not affected by IL-1β/OB/3-MA. **(A)** Molecular structure of OB. **(B)** The viability of chondrocytes treated with IL-1β (5 ng/ml), OB (160 μM), 3-MA (5 mM) for 24 h was measured by a CCK-8 kit. Data were presented as means ± SD (n = 3). ns, no significance.

The viability of chondrocytes treated with IL-1β or OB or 3-MA for 24 h was determined by a CCK-8 kit. The selection of time duration of the above reagent on chondrocytes is based on previous studies by us and others (5, 14–17). The chemical structure of OB was showed in **Figures 1A, B** demonstrated that the viability of chondrocytes was not significantly affected when cells were administration with 5 ng/ml IL-1β or 160 μM OB or 5 mM 3-MA for 24 h (*p* > 0.05).

### OB upregulated the anabolism in IL-1β–induced chondrocytes

**Figure 2:**
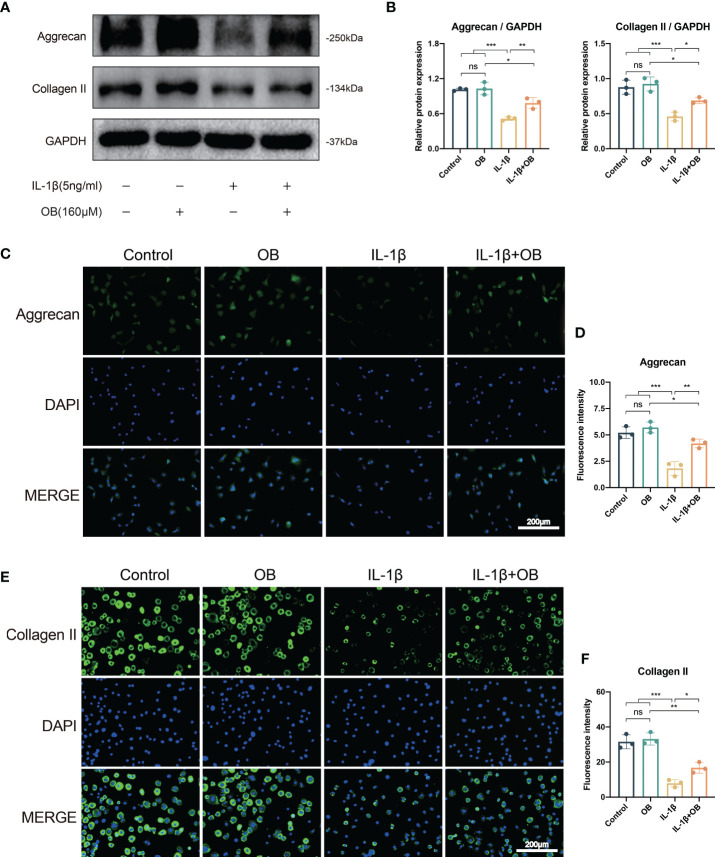
OB upregulated the anabolism in IL-1β–induced chondrocytes. Chondrocytes were treated with IL-1β (5 ng/ml) or/and OB (160 μM) for 24 h. **(A)** Western blot analysis and **(B)** quantitative data for the key anabolic-related proteins (Aggrecan, Collagen II). **(C, E)** Immunofluorescence staining and **(D, F)** quantitative data of the expression levels of Aggrecan and Collagen II. Data were presented as means ± SD (n = 3). ns, no significance; *p < 0.05; **p < 0.01; ***p < 0.001.

Our preliminary experimental results confirmed that the concentration of OB at 160 μM has a more obvious effect in protecting the anabolism and catabolism in IL-1β–induced chondrocytes, therefore, we selected the concentration of OB at 160 µM for the following experiment. As shown in **Figures 2A, B**, western blot analysis revealed that IL-1β decreased the expression level of key anabolic-related phenotypes, Aggrecan and Collagen II. While the administration of 160 μM OB increased the expression level of the above two proteins in IL-1β–induced chondrocytes. Besides, the data of western blot about the protein levels of Aggrecan and Collagen II were verified by immunofluorescence staining, as the decreased protein expression of Aggrecan and Collagen II in the cytoplasm induced by IL-1β were upregulated after the OB treatment (**Figures 2C–F**).

### OB downregulated the catabolism in IL-1β–induced chondrocytes

**Figure 3:**
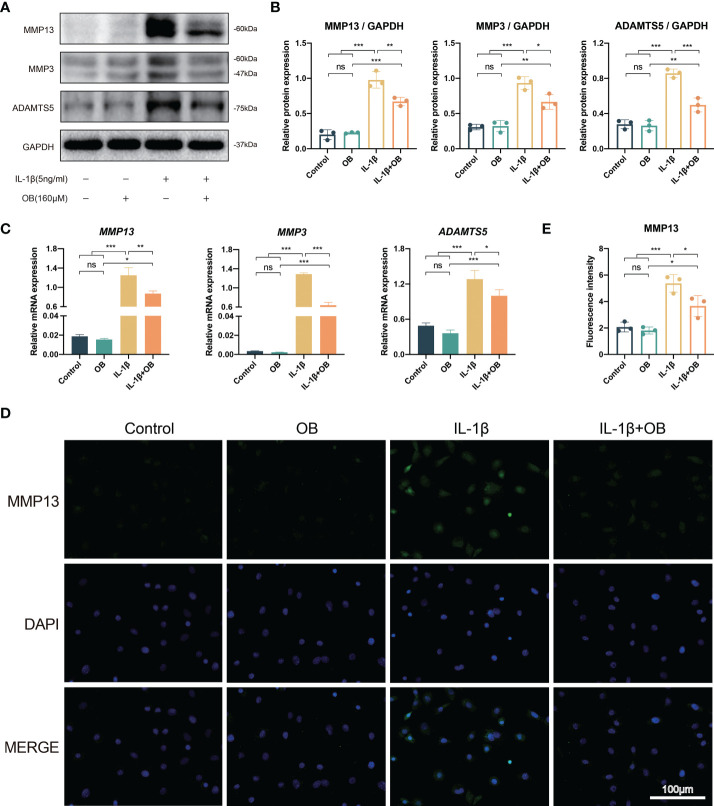
OB downregulated the catabolism in IL-1β–induced chondrocytes. Chondrocytes were administered with IL-1β (5 ng/ml) or/and OB (160 μM) for 24 h. **(A)** Western blot analysis and **(B)** quantitative data for the key catabolic-related proteins (MMP13, MMP3, and ADAMTS5). **(C)** Quantitative analysis of the catabolic-related (MMP13, MMP3, and ADAMTS5) mRNA expression by using RT-qPCR analysis. **(D)** Immunofluorescence staining and **(E)** quantitative data of the protein expression of MMP13. Data were presented as means ± SD (n = 3). ns, no significance; *p < 0.05; **p < 0.01; ***p < 0.001.

In **Figures 3A, B**, western blot data showed that the control group and the OB group exhibited a low expression level of catabolic-related proteins, such as MMP3, MMP13, and ADAMTS5. However, the IL-1β group showed an overexpression of above proteins, while IL-1β + OB group exhibited a decreased expression, which meant that OB downregulated the expression of the above proteins in IL-1β–induced chondrocytes. In addition, RT-qPCR analysis illustrated that the increased mRNA levels of gene *MMP3, MMP13, and ADAMTS5* induced by IL-1β could be downregulate by OB treatment (**Figure 3C**). Immunofluorescence staining also verified the results above, as the fluorescence intensity of MMP13 in the cytoplasm was increased in the IL-1β group and decreased in the IL-1β + OB group (**Figures 3D, E**).

### OB attenuated the inflammatory responses in IL-1β–induced chondrocytes

**Figure 4:**
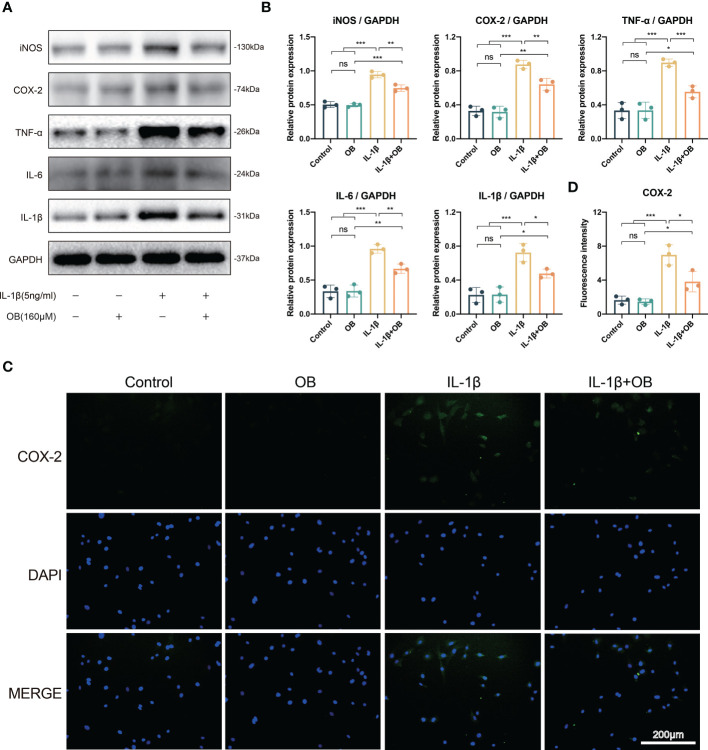
OB attenuated the inflammatory responses in IL-1β–induced chondrocytes. Chondrocytes were administered with IL-1β (5 ng/ml) or/and OB (160 μM) for 24 h. **(A)** Western blot analysis and **(B)** quantitative data for inflammatory proteins (iNOS, COX-2, TNF-α, IL-6, and IL-1β) in chondrocytes. **(C)** Immunofluorescence staining and **(D)** quantitative analysis of the expression levels of COX-2. Data were presented as means ± SD (n = 3). ns, no significance; *p < 0.05; **p < 0.01; ***p < 0.001.

To explore whether OB possessed the anti-inflammatory effect in OA chondrocytes, we performed the western blot and detected the pro-inflammatory markers, such as inducible nitric oxide synthase (iNOS), COX-2, TNF-α, IL-6, and IL-1β. As exhibited in **Figures 4A, B**, there is a higher expression level of the pro-inflammatory proteins in the IL-1β group than that of in the control group and the OB group. However, OB mitigated the inflammatory responses, as the treatment of OB in IL-1β–induced chondrocytes exhibited a decreased expression level of the pro-inflammatory proteins. Immunofluorescence staining also verified that the increased expression of COX-2 in IL-1β–induced chondrocytes could be inhibited by OB treatment (**Figures 4C, D**).

### OB suppressed the activation of PI3K/AKT/mTOR pathway in IL-1β–induced chondrocytes

**Figure 5:**
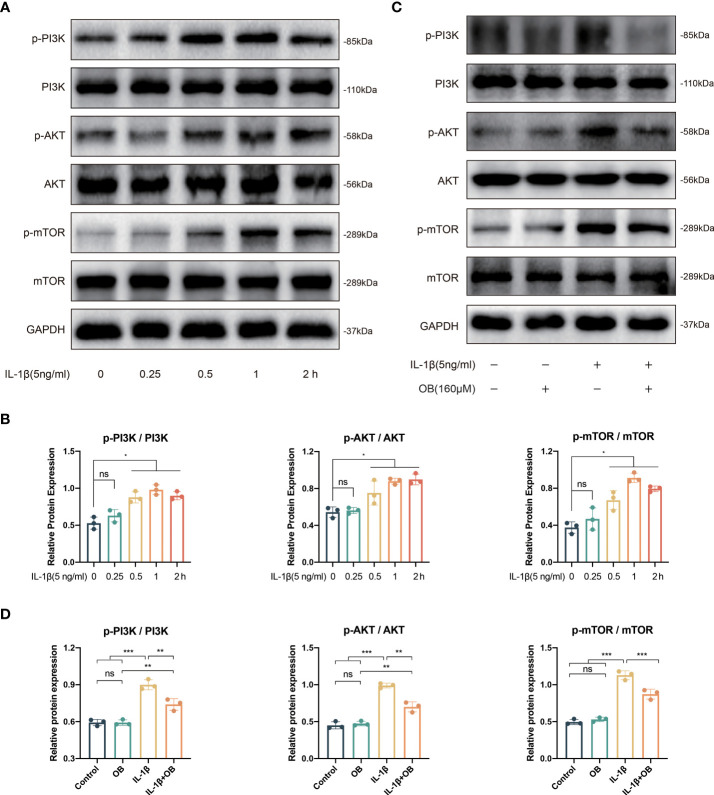
IL-1β triggered the early activation of PI3K/AKT/mTOR signaling pathway and OB inhibited the PI3K/AKT/mTOR signals in IL-1β–induced chondrocytes. **(A)** Western blot and **(B)** quantitative analysis for proteins associated with the PI3K/AKT/mTOR pathway in chondrocytes stimulated with 5 ng/ml IL-1β at different time durations (0, 0.25, 0.5, 1, and 2h). **(C)** Western blot analysis and **(D)** quantitative data the above proteins in chondrocytes which were administered with IL-1β (5 ng/ml) for 1 h or/and OB (160 μM) for 24 h. Data were presented as means ± SD (n = 3). ns, no significance; *p < 0.05; **p < 0.01; ***p < 0.001.

The activation of PI3K/AKT/mTOR pathway is closely related to impaired autophagy process, as well as the inflammatory responses. As shown in **Figures 5A, B**, IL-1β triggered the early activation of PI3K/AKT/mTOR signaling pathway, as the key proteins at the node of this signaling pathway such as phospho-PI3K (p-PI3K), p-AKT, phospho-mTOR (p-mTOR) were increased after the stimulation of IL-1β. Besides, the activation process induced by IL-1β was time-dependent and peaked at almost 1 h. Therefore, we selected the strongest stimulus time point of 1 h to explore the effect of OB on the PI3K/AKT/mTOR signaling pathway. In **Figures 5C, D**, we observed that the activation of the PI3K/AKT/mTOR pathway could be inhibited by OB in IL-1β–induced chondrocytes.

### OB rescued the impaired autophagy process in IL-1β–induced chondrocytes

**Figure 6:**
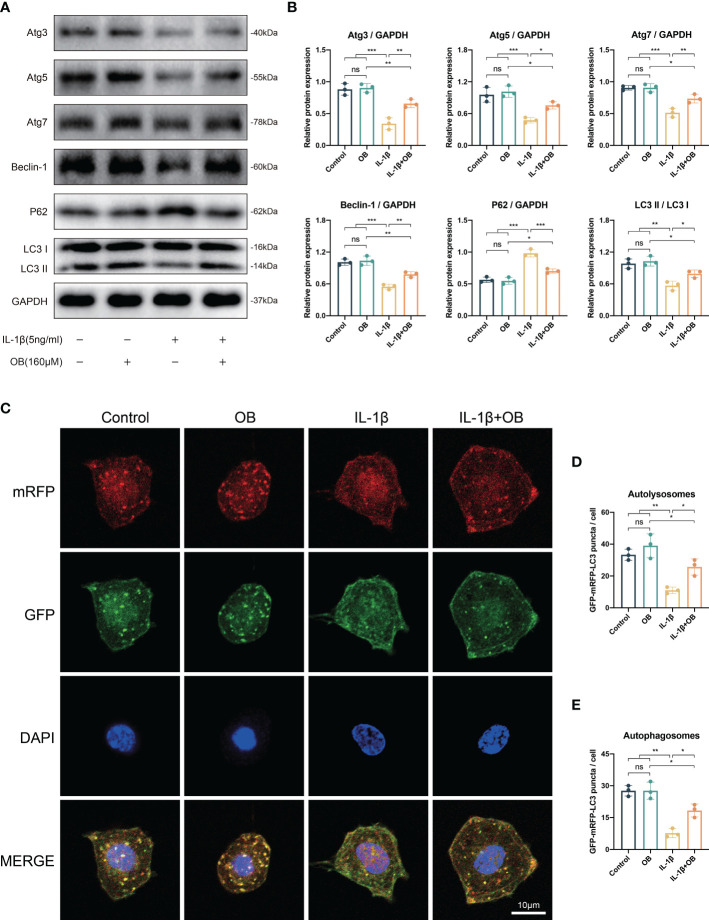
OB rescued the impaired autophagy process in IL-1β–induced chondrocytes. Chondrocytes were administered with IL-1β (5 ng/ml) or/and OB (160 μM) for 24 h. **(A)** Western blot and **(B)** quantitative analysis showed the impaired autophagy induced by IL-1β could be rescued by the treatment of OB (160 μM). **(C)** Chondrocytes were transfected with tandem GFP-RFP-LC3 adenovirus, and the strength of autophagic flux was captured with a confocal microscope. **(D, E)** Quantitative analysis of autolysomes (red puncta) and autophagosomes (yellow puncta) among groups. Data were presented as means ± SD (n = 3). ns, no significance; *p < 0.05; **p < 0.01; ***p < 0.001.

As shown in **Figures 6A, B**, the autophagy process was at a relatively high level in the control group and the OB group compared with that in the IL-1β group, as IL-1β decreased the autophagy positive regulatory proteins, such as Atg3, Atg5, Atg7, Beclin-1, and LC3 I/II, while increased the autophagy negative regulatory protein, P62. However, the damaged autophagy process could be reversed in the IL-1β + OB group, as the administration of OB in the IL-1β–induced chondrocytes upregulated the above autophagy positive regulator and downregulated the autophagy negative regulator. Besides, in autophagy flux detection experiments, the mRFP was applied to label and track the LC3, the yellow puncta represented the autophagosomes, while the red puncta reflected the autolysosomes. As shown in **Figures 6C–E**, we also observed that IL-1β led to the decreased autophagic flux compared with the control group and the OB group; however, an enhanced autophagic flux was observed in IL-1β–induced chondrocytes after the OB treatment.

### Autophagy inhibitor 3-MA reversed the effect of OB on autophagy process

**Figure 7:**
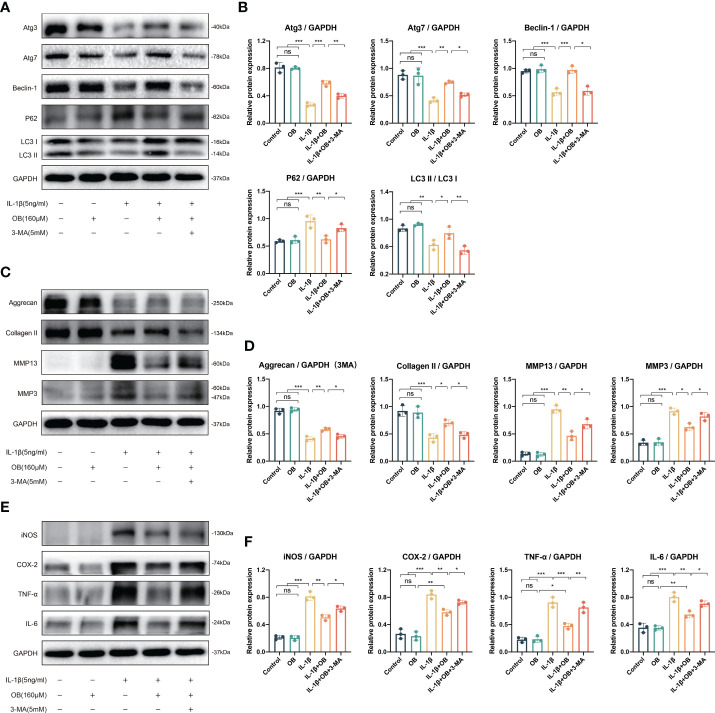
3-MA reversed the anti-cartilage degradation and the anti-inflammatory effects of OB in IL-1β–induced chondrocytes. Chondrocytes were administered with IL-1β (5 ng/ml) or/and OB (160 μM) or/and 3-MA (5 mM)for 24 h. **(A)** Western blot analysis and **(B)** quantitative data for autophagy-related proteins (Atg3, Atg7, Beclin-1, P62, and LC3 I/II) in chondrocytes. **(C)** Western blot analysis and **(D)** quantitative data for anabolic and catabolic-related proteins (Aggrecan, Collagen II, MMP3, and MMP13). **(E)** Western blot analysis and **(F)** quantitative data for inflammatory proteins (iNOS, COX-2, TNF-α, and IL-6). Data were presented as means ± SD (n = 3). ns, no significance; *p < 0.05; **p < 0.01; ***p < 0.001.

3-MA is a commonly used autophagy inhibitor (18, 19). As shown in **Figures 7A, B**, the increased autophagy positive regulatory proteins (such as Atg3, Atg7, Beclin-1, and LC3 I/II) and decreased autophagy negative regulatory protein (P62) by OB were reversed after the employment of 3-MA, indicating that the enhanced autophagy process by OB was suppressed after the 3-MA treatment.

### Autophagy inhibitor 3-MA reversed the anti-cartilage degradation and the anti-inflammatory effects of OB in IL-1β–induced chondrocytes

In order to clarify whether the anti-cartilage degradation and anti-inflammatory effects of OB was attribute to the mechanism of regulating the autophagy process. 3-MA was employed to inhibit the autophagy process in the experiment. The data in **Figures 7C, D** demonstrated that 3-MA inhibited the anabolism-enhancing effect of OB in IL-1β-induced chondrocytes. Meanwhile, 3-MA could reverse the catabolism-inhibitory effect of OB in IL-1β-induced chondrocytes. In addition, the anti-inflammatory effect of OB in IL-1β–induced chondrocytes was also reversed in the IL-1β + OB + 3-MA group compared with that in the IL-1β + OB group (**Figures 7E, F**). Thus, we concluded that the anti-cartilage degradation and the anti-inflammatory effects of OB in IL-1β–induced chondrocytes could be attributed to the enhancing autophagy process.

### OB ameliorated cartilage destruction in DMM–induced mice OA model

**Figure 8:**
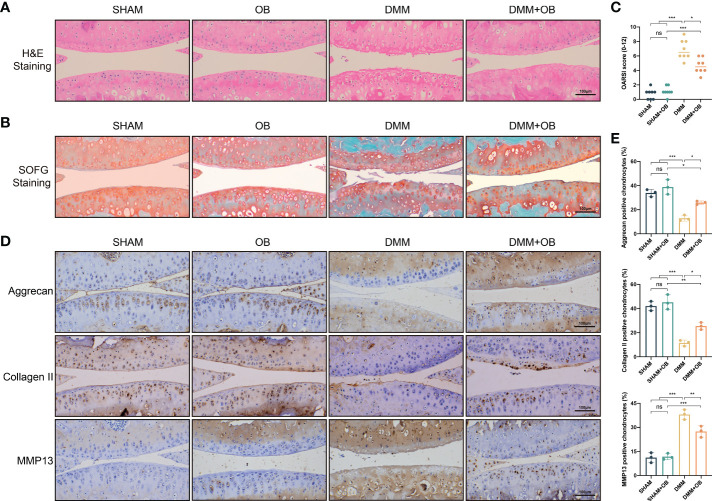
OB alleviated cartilage destruction in DMM–induced mice OA model. **(A)** H&E staining and **(B)** SOFG staining of the mice knee articular cartilage. **(C)** Quantitative analysis of the degree of cartilage destruction among groups by the OARSI scoring system (n=8). **(D)** Immunohistological staining and **(E)** quantitative analysis of anabolic and catabolic-related proteins (Aggrecan, Collagen II, and MMP13) (n=3). Data were presented as means ± SD (n = 3). ns, no significance; *p < 0.05; **p < 0.01; ***p < 0.001.

The *in vivo* experiment was to explore the effect of OB in mice knee cartilage. The DMM method was employed to establish the OA cartilage model. H&E and SOFG staining showed that the DMM group characterized by OA features, such as unevenness, thinning, and cracking of the cartilage surface, and reduction of chondrocytes compared with the SHAM group and the SHAM + OB group. However, we observed a chondroprotective effect of OB in the DMM + OB group, as cartilage damage was alleviated after the OB treatment (**Figures 8A, B**). Besides, the chondroprotective effect of OB was verified by the OARSI score system which reflected the severity of cartilage damage (**Figure 8C**). In addition, the detection of key proteins of anabolism and catabolism in chondrocytes was also performed *in vivo* experiments. IHC staining (**Figures 8D, E**) exhibited a decreased expression of anabolic-related proteins (Aggrecan, Collagen II) and an increased expression of catabolic-related protein (MMP13) in the DMM group compared with the SHAM group and the SHAM + OB group, while the changes were reversed in the DMM + OB group, which was consistent with results illustrated *in vitro* experiment. Collectively, the above data proved that OB prosses the property of protecting articular cartilage in DMM-induced OA *in vivo*.

## Discussion

Articular cartilage, which is consist of chondrocytes and ECM, is a crucial component of the joint. The degradation or destruction of cartilage mediated by factors of the chondrocytes or the ECM is an initiating factor leading to OA. Since chondrocytes are considered to be the only cell type in cartilage, the functional state of chondrocytes is critical for cartilage homeostasis. A good balance of anabolism and catabolism in chondrocytes can maintain the normal morphology of articular cartilage. If the balance is disturbed, it will lead to the degradation of the ECM, which will trigger the destruction of cartilage and the occurrence of OA. Therefore, factors such as inflammatory response, trauma, mechanical load, and aging, etc. are high risk factors for OA because of their contribution to the imbalance state of cartilage metabolism. Aggrecan and Collagen II are the main two key indicators of chondrocyte anabolism. Aggrecan is a proteoglycan with anti-compressive property that plays a key role in cartilage structure and joint function (20). Meanwhile, Collagen II in cartilage with tensile strength properties due to its special network structure (21). Aggrecanase-mediated degradation of Aggrecan or Collagenase-mediated degradation of Collagen II are important signal of early OA (22, 23). The main structure of the ECM is composed of Aggrecan and Collagen II, which can maintain the normal physiological function of articular cartilage. Loss of Aggrecan and Collagen II contributes to the OA progression. As the proteins that reflect the catabolism, such as MMP3, MMP13, and ADAMTS5, play a crucial role in cartilage degradation. MMP13, together with MMP3, is an efficient catabolic enzyme in chondrocyte catabolism and can degrade Collagen II, Collagen IV, Collagen IX and Aggrecan. ADAMTS5 is the main aggrecanase in chondrocyte and one of the important members of ADAMTS family that degrades aggrecan. Therefore, chondrocyte anabolism and catabolism are crucial to the ECM of cartilage and maintain the dynamic balance of the ECM. Any factors that lead to an imbalance of anabolism and catabolism in chondrocytes will contribute to the degradation of cartilage and the occurrence of OA. On the contrary, studies have revealed that measures to maintain metabolic balance improve OA.

For the pathogenic mechanism of OA, the imbalance of inflammatory response is considered to be one of the main mechanisms (24). Studies have confirmed that chronic inflammation of the surrounding tissue is also an important characteristic of OA (25). In addition, the activation of inflammatory signaling pathways such as MAPK or NF-κB or the release of a large number of pro-inflammatory cytokines (iNOS, COX-2, TNF-α, IL-6, and IL-1β) can trigger the imbalance of anabolism and catabolism in chondrocytes, leading to the occurrence of cartilage degeneration and presence of clinical symptoms of OA such as pain and swelling (26–28). Basic experimental studies have shown that measures to inhibit inflammatory responses can restore the balance of anabolism and catabolism in chondrocytes and alleviate cartilage damage (29). Besides, clinical evidence has confirmed that the administration of non-steroidal anti-inflammatory drugs can effectively relieve clinical symptoms in patients with OA (30). Oroxin B (OB), a bioactive substance extracted from traditional Chinese herbal medicine *Oroxylum indicum (L.) Vent (* 8), was provided with anti-inflammation property by inhibiting COX-2/VEGF, PTEN/PI3K/AKT, MAPK, and NF-κB signaling pathways, whereas seldom research was investigated about the effect of OB on OA (9, 10). The data of our study showed that OB down-regulated the expression of inflammatory proteins and reversed the disorder of anabolism and catabolism in IL-1β–induced chondrocytes, demonstrating the anti-inflammatory and articular cartilage protective properties of OB.

The survival state of chondrocytes is crucial to their physiological functions. Autophagy can remove harmful substances to cells and is considered as an important protective mechanism in OA (31). An impaired regulation of autophagy process could lead to dysfunction or death of chondrocytes, then abnormal anabolism and catabolism, degradation of ECM, and occurrence of OA (32). The PI3K/AKT/mTOR pathway is closely related to the regulation of autophagy. mTOR, as a downstream key node of the PI3K/AKT/mTOR pathway, could regulate the autophagic signaling pathway in a negatively manner (33). Therefore, measures of suppressing activation of the PI3K/AKT/mTOR pathway can promote autophagy and alleviate OA (13). Besides, the upregulated expression level of autophagy positive regulatory proteins, such as Atg3, Atg5, Atg7, and Beclin-1 reflect enhancement of autophagy process, while the autophagy negative regulatory protein, P62, indicates impaired autophagy. In addition, the increase of the ratio of LC3 II to LC3 I also indicates the upregulation of autophagy. The down-regulated autophagy process leads to decreased anabolism and enhanced catabolism in chondrocytes and exacerbates the development of OA (34). In our study, the reduced autophagy process in chondrocytes induced by IL-1β was restored after the administration of OB. However, the autophagy-enhancing effect of OB on IL-1β–induced chondrocytes was partially blocked by the autophagy inhibitor 3-MA, implying that OB protects articular cartilage through the autophagy pathway.

Inflammation and autophagy are mutually causal and can regulate each other. Autophagy is involved in the regulation of inflammatory process in OA. Previous study revealed that activation of autophagy alleviates OA chondrocytes inflammation, as the enhanced autophagy decreased the gene expression of IL-1β, IL-6, and TNF-α (16). Besides, the inflammatory response in OA also relates to the regulation of autophagy. Various studies have confirmed that inflammatory factors can lead to the attenuation of autophagy (35, 36). The reciprocal regulation of inflammation and autophagy mentioned above was also confirmed in our study, as inflammatory cytokines, IL-1β, decreased the autophagic signaling in mice chondrocytes, whereas the autophagy inhibitor 3-MA enhanced the inflammatory response by inhibiting the autophagy process. Furthermore, inhibition of the autophagic process by 3-MA abolished the protective effect of OB on cartilage, indicating that the protecting role of OB on OA was attributed to the regulation of autophagy.

**Figure 9:**
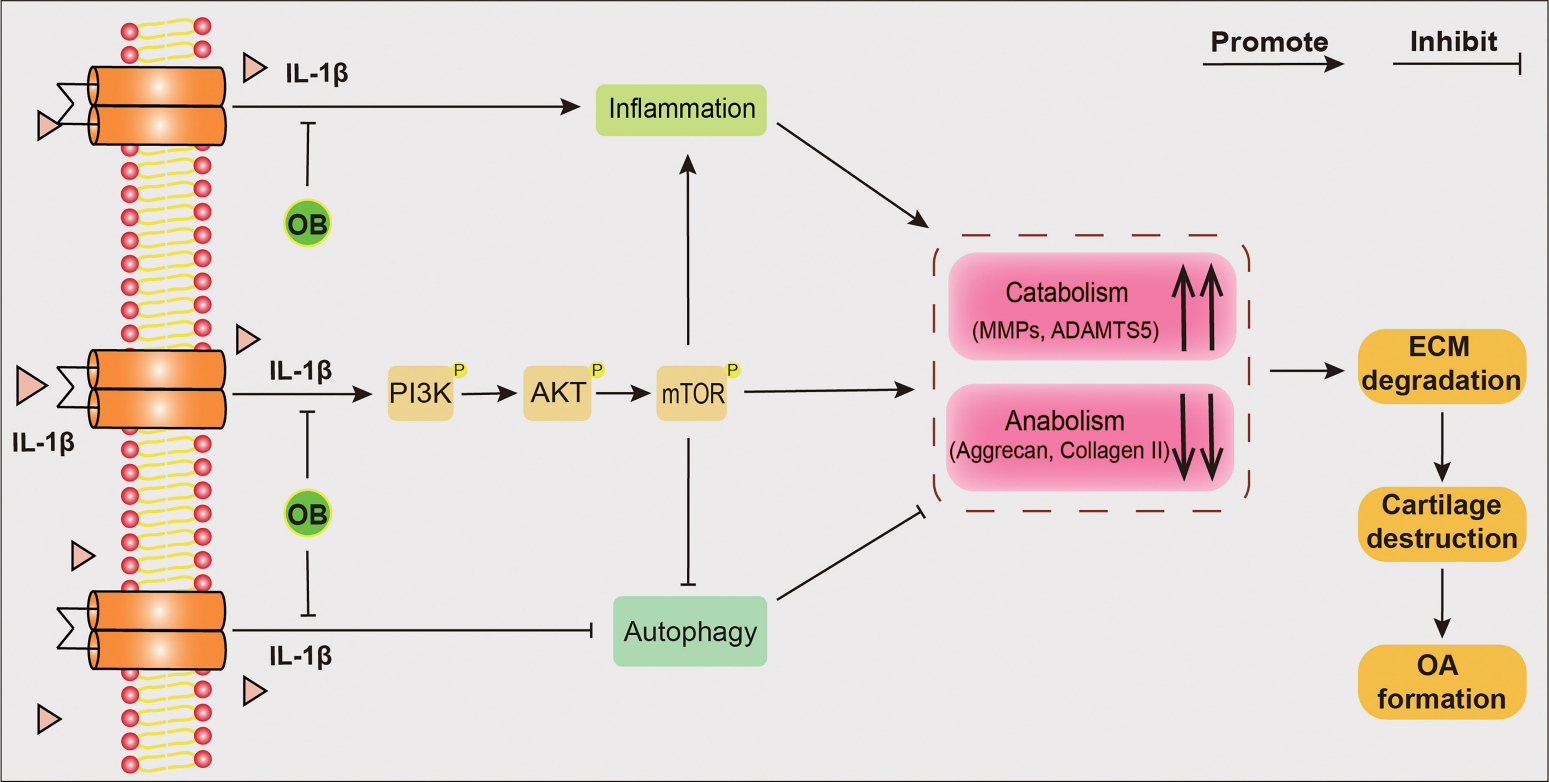
Schematic diagram of the chondroprotective effect of OB on mice OA. IL-1β–induced OA chondrocytes were employed in *in vitro* experiments. The anabolism–inhibiting, catabolism–enhancing, and inflammation-promoting effects induced by IL-1β could be reversed after the treatment of OB. Mechanistically, OB inhibted the activation of PI3K/AKT/mTOR signaling and restored the reduced autophagy process in IL-1β induced chondrocytes. However, 3-MA, an autophagy inhibitor, abolished the protective effect of OB on cartilage, indicating that OB plays a protecting role in articular cartilage through the autophagy process.

In conclusion, as shown in **Figure 9**, the *in vitro* experiments demonstrated that OB protected the articular cartilage by inhibiting the inflammatory response and the PI3K/AKT/mTOR signaling pathway and enhancing the autophagy process. Additionally, 3-MA abolished the chondroprotective effect of OB on OA. The *in vivo* experiments revealed that the degradation of cartilage and the imbalance of anabolism and catabolism in chondrocytes induced by DMM surgery were reversed by the administration of OB, suggesting that OB might be a promising agent for OA treatment. However, some shortcomings were still existed in our study. The cartilage protecting mechanism of OB was only verified in mouse *in vitro* experiments, not confirmed in *in vivo* experiments. In addition, the direct targets of the therapeutic effect of OB on OA need to be further explored.

## Data availability statement

The original contributions presented in the study are publicly available. This data can be found here: https://www.jianguoyun.com/p/DVdvZmkQnbaACxiLnugEIAA.

## Ethics statement

The animal study was reviewed and approved by Ethics Committee on Animal Experimentation of Tongji Hospital, Tongji Medical College, Huazhong University of Science and Technology.

## Author contributions

RL: Conceptualization; Data curation; Methodology; Writing – review &editing. ZH: Data curation; Formal analysis; Methodology. WZ: Data curation; Formal analysis; Methodology. YW: Methodology; Validation. PC: Methodology; Validation ZL: Methodology; Validation. XY: Methodology; Data curation. FG: Data curation; Validation. Role/editing – original draft. HY: Conceptualization; Role/editing – original draft. AC: Conceptualization; Writing – review & editing; Supervision. WH: Conceptualization; Data curation; Funding acquisition; Project administration; Writing – review & editing. All authors contributed to the article and approved the submitted version.

## Funding

This study was supported by Hubei Provincial Natural Science Foundation of China (2016CFB493) and Wuhan Youth Chenguang Program of Science and Technology (2014070404010220).

## Acknowledgments

We sincerely appreciate all the participants who provided support in the study.

## Conflict of interest

The authors declare that the research was conducted in the absence of any commercial or financial relationships that could be construed as a potential conflict of interest.

## Publisher’s note

All claims expressed in this article are solely those of the authors and do not necessarily represent those of their affiliated organizations, or those of the publisher, the editors and the reviewers. Any product that may be evaluated in this article, or claim that may be made by its manufacturer, is not guaranteed or endorsed by the publisher.

## Abbreviations

OA, osteoarthritis; OB, Oroxin B; IL-1β, interleukin-1β; Collagen II, type II collagen; MMP3, matrix metallopeptidase 3; MMP13, matrix metallopeptidase 13; ADAMTS5, a disintegrin and metalloproteinase with thrombospondin motifs 5; ECM, extracellular matrix; MAPK, mitogen-activated protein kinase; NF-κB, nuclear factor-kappa B; FBS, foetal bovine serum; cDNA, complementary DNA; RT-qPCR, quantitative real-time polymerase chain reaction; CCK-8, cell counting kit-8; 3-MA, 3-methyladenine; BSA, bovine serum albumin; COX-2, cyclooxygenase-2; PI3K, phosphoinositide 3-kinase; DMM, destabilized medial meniscus; iNOS, inducible nitric oxide synthase; OARSI, Osteoarthritis Research Society International; H&E, hematoxylin plus eosin; SOFG, safranin O/fast green; IHC, immunohistochemistry.
